# Andy’s Algorithms: new automated digital image analysis pipelines for FIJI

**DOI:** 10.1038/s41598-017-15885-6

**Published:** 2017-11-16

**Authors:** Andrew M. K. Law, Julia X. M. Yin, Lesley Castillo, Adelaide I. J. Young, Catherine Piggin, Samuel Rogers, Catherine Elizabeth Caldon, Andrew Burgess, Ewan K. A. Millar, Sandra A. O’Toole, David Gallego-Ortega, Christopher J. Ormandy, Samantha R. Oakes

**Affiliations:** 1 Garvan Institute of Medical Research and the Kinghorn Cancer Centre, 384 Victoria Street, Darlinghurst, NSW 2010 Australia; 2 St. Vincent’s Clinical School, UNSW Sydney, Darlinghurst, Victoria Street, NSW 2052 Australia; 3 ANZAC Research Institute, University of Sydney, 3 Hospital Road, Concord, NSW 2139 Australia; 4 Department of Anatomical Pathology, South Eastern Area Laboratory Service, St George Hospital, Grey St. Kogarah, Kogarah, 2217 Australia; 5 School of Medical Sciences, UNSW Sydney, Kensington, NSW 2033 Australia; 6 School of Medicine and Health Sciences, Sydney Western University, Campbelltown, NSW 2560 Australia; 7 Australian Clinical Labs, 112/14 Lexington Drive, Bella Vista, NSW 2153 Australia

## Abstract

Quantification of cellular antigens and their interactions via antibody-based detection methods are widely used in scientific research. Accurate high-throughput quantitation of these assays using general image analysis software can be time consuming and challenging, particularly when attempted by users with limited image processing and analysis knowledge. To overcome this, we have designed Andy’s Algorithms, a series of automated image analysis pipelines for FIJI, that permits rapid, accurate and reproducible batch-processing of 3,3**′**-diaminobenzidine (DAB) immunohistochemistry, proximity ligation assays (PLAs) and other common assays. Andy’s Algorithms incorporates a step-by-step tutorial and optimization pipeline to make batch image analysis simple for the untrained user and adaptable across laboratories. Andy’s algorithms provide a simpler, faster, standardized work flow compared to existing programs, while offering equivalent performance and additional features, in a free to use open-source application of FIJI. Andy’s Algorithms are available at GitHub, publicly accessed at https://github.com/andlaw1841/Andy-s-Algorithm.

## Introduction

Immune-based antigen recognition techniques and quantitative image analysis in cells and tissues are widely used in scientific research. Immunohistochemistry (IHC) is used for the detection and quantification of target antigens in formalin-fixed paraffin embedded tissue sections for a variety of research applications and human cancer phenotyping. For example, the expression of estrogen receptor (ER), progesterone receptor (PR), human epidermal growth factor receptor 2 (HER2), proliferation marker protein Ki67 and epidermal growth factor receptor (EGFR) is used for prognostication in patients diagnosed with breast cancer and informs treatment decisions^1–4^. Routine IHC employs the use of thermodynamically and chemically stable 3,3**′**-diaminobenzidine (DAB), which oxidizes in the presence of hydrogen peroxide or peroxidase conjugated antibodies and hemoglobin to produce a dark brown precipitate. The DAB+ precipitate is stable and permits antigen identification in tissues *in situ* through bright-field light microscopy^5,6^. Research applications of DAB+ IHC include defining cellular phenotype within tissues, investigating the spatiotemporal expression patterns of proteins in tissues and investigating how proteins change with exogenous and endogenous stimuli. A new technology, based on antibody-antigen recognition, is the proximity ligation assay (PLA) that detects sub-cellular single molecule protein interactions in fixed cells via high-resolution fluorescence microscope^7^. The PLA uses a combination of antibody-based detection methods coupled to rolling circle DNA synthesis to create fluorescent foci when two target proteins are in close proximity. This method is often combined with a nuclear stain and/or a cytoplasmic stain to aid analysis. Quantification of images produced from these techniques can be performed manually or with the assistance of commercially available image analysis programs.

Manual quantification of large numbers of tissue samples is time-intensive and subject to cognitive bias, resulting in variable and erroneous results. Computer-aided analysis with open source or commercial software overcomes cognitive bias and provides more accurate methods of quantitative image analysis for the evaluation of specific protein proximity, abundance, and interaction. The pioneer for open-source image processing and analysis was the Java-based NIH Image for Macintosh developed in the mid-late 1990s, which was later replaced by ImageJ^8^. A newer open source package based on ImageJ, called FIJI, runs on both Windows and Macintosh PC platforms and incorporates a large number of bundled plugins for scientific image processing and analysis, and is widely used by the scientific community^9^. Other image processing and analysis programs include ImmunoRatio^10^, and ilastik^11^, Icy^12^, Daime^13^, BlobFinder^14^, CellProfiler^15^, MATLAB, MetaMorph, Duolink® ImageTool and Imaris (Supp. Table 1). The latter four are commercially available programs requiring a subscription fee and additional application-specific plug-ins. Although these general image analysis programs are both powerful and versatile, untrained users find it challenging to understand each program and to create pipelines for accurate high throughput image analysis of specific assays. This ‘usability issue’ of bio-imaging software has become an issue of great importance to biologists and developers alike, where software is developed but not widely adopted by a broader scientific community^16^.

Our laboratory has used both manual and computer aided automated analysis techniques for image analysis of cells and tissues *in vitro* and *in vivo* ^17–20^. We have confronted several challenges with image analysis, including overcoming expected variations in staining of mouse tumors and tissues, poor discrimination between browns and blues in images, uneven illumination in photomicrographs and the creation of pipelines for unbiased high-throughput automatic analyses yielding reproducible results. For example, automatic imaging analysis of IHC specimens requires sufficient contrast and color discrimination between DAB+ (dark brown) and negative hematoxylin (dark blue) counterstain^21^. Additionally, there are currently no open-access all-in-one batch image analysis pipelines for quantification of DAB+ IHC or PLAs. To deal with these challenges we developed a package named Andy’s Algorithms, a series of four separate all-in-one automated image analysis pipelines for common laboratory antigen recognition immunoassays, embedded with step-by-step tutorials and designed for FIJI^9^.

## Results

### A pipeline for quantification of DAB+ immunohistochemistry

**Figure 1:**
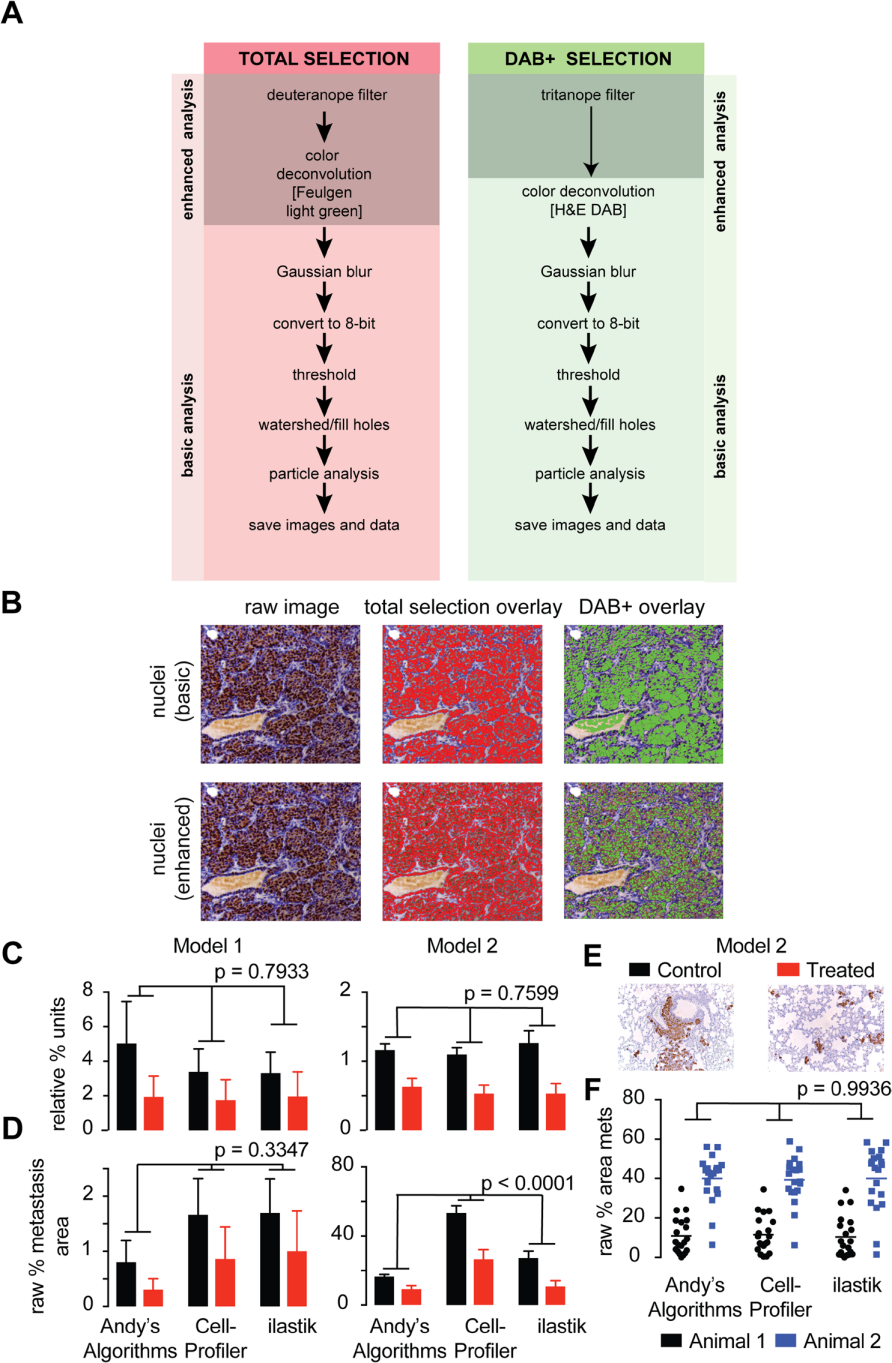
A new pipeline for image quantification of DAB+ IHC. (**A**) Flow chart depicting the image processing steps within the DAB+ algorithm for the selection of all (hematoxylin and DAB+) and positive cells (DAB+ only). The basic algorithm is used when there is appropriate discrimination between blues and browns in any given IHC image, and the enhanced algorithm should be used when poor discrimination between blues and browns is apparent (**B**) Representative IHC images (left panels) showing poor discrimination between blues and browns and the selection overlays for total cells (middle panels) and DAB+ cells (right panels) for both the basic and enhanced DAB+ algorithm. Bar graphs depicting the normalized (**C**) and raw (**D**) metastatic area calculated by Andy’s DAB+ algorithm and compared to CellProfiler and ilastik of metastatic area of the lungs of mice bearing xenografts from two different breast cancer models^22^. An average measurement from 3 mice was calculated from the average of 10 images is depicted. Representative DAB+ IHC images used in the analysis in C and D is shown in (**E**). (**F**) Bar graphs depicting the calculated raw metastatic area in individual images (20 per mouse) in the lungs of two mice (blue and black) corrected for uneven illumination and white background calculated with Andy’s DAB+ algorithm and compared to CellProfiler and ilastik using data from^22^. ANOVA p-value tests variance across algorithms.

Andy’s DAB+ IHC pipeline was developed as a batch analysis tool to quantitate metastatic deposits in the lungs of mice bearing breast cancer xenografts^22^. The DAB+ image analysis algorithm includes an all-in-one tutorial, optimization and analysis pipeline for particle analysis (number, percentage positive, area and intensity) for immunohistochemistry (IHC) sections employing DAB+ as a detection method. The DAB+ IHC algorithm first provides the user with a choice to optimize the parameters in the pipeline on a set of 3–5 test images, prior to running the batch image analysis on a larger set of images. The image processing steps contained within the pipeline are illustrated in Fig. 1A and illustrated in Supp. Figure 1 and are designed specifically to detect regions of brown (DAB+) and blue (hematoxylin).

Users may select a basic or enhanced pipeline for DAB+ and total ROI selection. In the enhanced pipeline, a color blindness filter is applied to a raw image to improve selection of regions of interest (ROIs) prior to the application of a color deconvolution filter to permit discrimination of browns and blues. A deuteranope filter is applied to simulate the vision of red-green color blindness in deutan individuals whom are unable to detect greens^23^. The deuteranope filter dilutes the color differences between blue and brown and permits total selection of nuclei and cytoplasm. A tritanope filter, which simulates tritan color blindness in individuals that lack blue photoreceptors^23^, is used to enhance the pigmentation differences between DAB+ brown and hematoxylin blue. A color deconvolution filter is used to further discriminate between colors based on their absorption spectra using either a Fuelgen light green filter (for total selection of nuclei or cytoplasm) or a H&E DAB filter (for selection of DAB+ nuclei and cytoplasm)^24^. Next a Gaussian blur is applied to the image and settings are optimized to smooth the image and reduce noise. The image is then converted to an 8-bit grey-scale image (Supp. Figure 1) before users are prompted to apply a threshold to convert the greyscale image to a binary image. A user may select a manual or automatic threshold, where a threshold is applied on all images based on either a manually set threshold value or an optimal threshold function using one of five main algorithms (Huang^25^, RenyiEntropy^26^ (or Li for the basic pipeline^27,28^), Otsu^29,30^, Shanbhag^31^, and Yen^32^). There are a total of 16 algorithms that can be used to apply an automatic threshold and the former have been selected for best performance in this pipeline. Each method applies a calculation to convert any given pixel in the 8-bit grey scale pixel into either a black (background) or white (ROI) pixel and is detailed on the ImageJ website at https://imagej.net/Auto_Threshold. A test montage image (Supp. Figure 1) is provided to allow users to visually inspect the output of each threshold calculation and determine the most appropriate threshold for ROIs in a cohort of images. The image is then converted to a binary image.

Once converted to a binary image, users are then provided with the option to apply binary processes such as watershed, fill holes, or edge exclusion before removing small and large particles. As the size of the cell, nuclei and cytoplasm varies widely depending on tissue type and staining method, users must determine the most appropriate lower and upper size exclusion parameters for a set of images. A table of recommended lower and upper size exclusion parameters for 10X, 20X and 40X objective magnifications for epithelial tissues (based on mouse mammary epithelium and human breast cancer cells) are provided in Supp. Table 2 as a guide. A scale can be set in SI units, if known, in the ‘set a scale’ dialog. A complete instructional guide of spatial calculations is provided on the ImageJ website at https://imagej.net/SpatialCalibration. If no scale is set the default unit is pixels. Image analysis is then performed on the ROIs in the test images and results output to a summary file in the target image folder. A glossary defining the output parameters in the summary spreadsheet is provided in Supp. Table 3. Finally, an overlay image of both the total and DAB+ selection for each image is produced so users can visually inspect whether the ROI selection is accurate for across a test cohort of images (Fig. 1B and Supp. Figure 1). Once the user is satisfied that the parameters are correct, the optimized parameters are entered for analysis of the entire cohort of samples (Fig. 1A). The pipeline above is suitable for most tissue subjected to DAB+ IHC including those specimens that have poor discrimination of brown and blue regions due to weak to strong DAB+ staining and hematoxylin staining for example (examples of poorly discriminated stains are provided in Fig. 1B). Users also have the option to use the basic version of the pipeline that excludes the application of deuteranope and tritanope filters, which may not be needed if the staining procedure produces images have good discrimination of DAB+ browns and blues (Supp. Figure 1).

### Andy’s DAB+ IHC pipeline provides a way to perform batch analysis of DAB+ IHC images and was benchmarked against the output of image analysis programs CellProfiler and ilastik

Andy’s DAB+ IHC pipeline was used to quantitate metastatic deposits in the lungs of mice bearing breast cancer xenografts^22^. Two human breast cancer xenograft models derived from human breast cancer cell lines (MDA-MB-468 cells, model 1 and MDA-MB-231 cells, model 2) were injected into the ducts of the mammary gland of 3–4 immune-compromised mice, a technique that promoted metastasis to lung. DAB+ IHC was performed was used to highlight the xenograft-derived metastatic deposits in the lungs using human specific antibodies to either high molecular weight cytokeratin (model 1) or Vimentin (model 2) (Fig. 1C and D). 20 images per animals were then analyzed with Andy’s DAB+ IHC algorithm and the output was validated with similar image analysis performed using CellProfiler and ilastik (Fig. 1C and D). Each platform employed different methods of user-adjusted threshold application and nuclei segmentation but employs the same color blindness and deconvolution filters could be used to discriminate colors in each image.

When the output of Andy’s algorithms was compared with that of CellProfiler and ilastik, all three programs were equivalent in their ability to discriminate the degree of change between treatment and control in the lungs of mice bearing two different breast tumor xenograft models, model 1 (Two-way ANOVA p = 0.7955) and model 2 (Two-way ANOVA p = 0.7599, Fig. 1C) when normalized to the lowest control sample. Some variation was observed in the baseline measurement of metastatic area in model 2 (Fig. 1D, Two-way ANOVA p < 0.0001). This may be due to uneven illumination of photomicrographs as shown in Fig. 1E that was tolerated better by Andy’s Algorithms DAB+ IHC pipeline via fine adjustment of threshold, size restriction, and edge exclusion included in the tutorial and analysis. When the same analysis was performed on images that had been corrected for background and uneven illumination, all three programs showed very good correlation with no statistical difference observed between groups (Fig. 1F) (Pearson’s R^2^ = 0.7816, Two-way ANOVA p = 0.99).

### Andy’s DAB+ IHC pipeline is useful for scoring tissue microarrays subjected to immunohistochemistry

**Figure 2:**
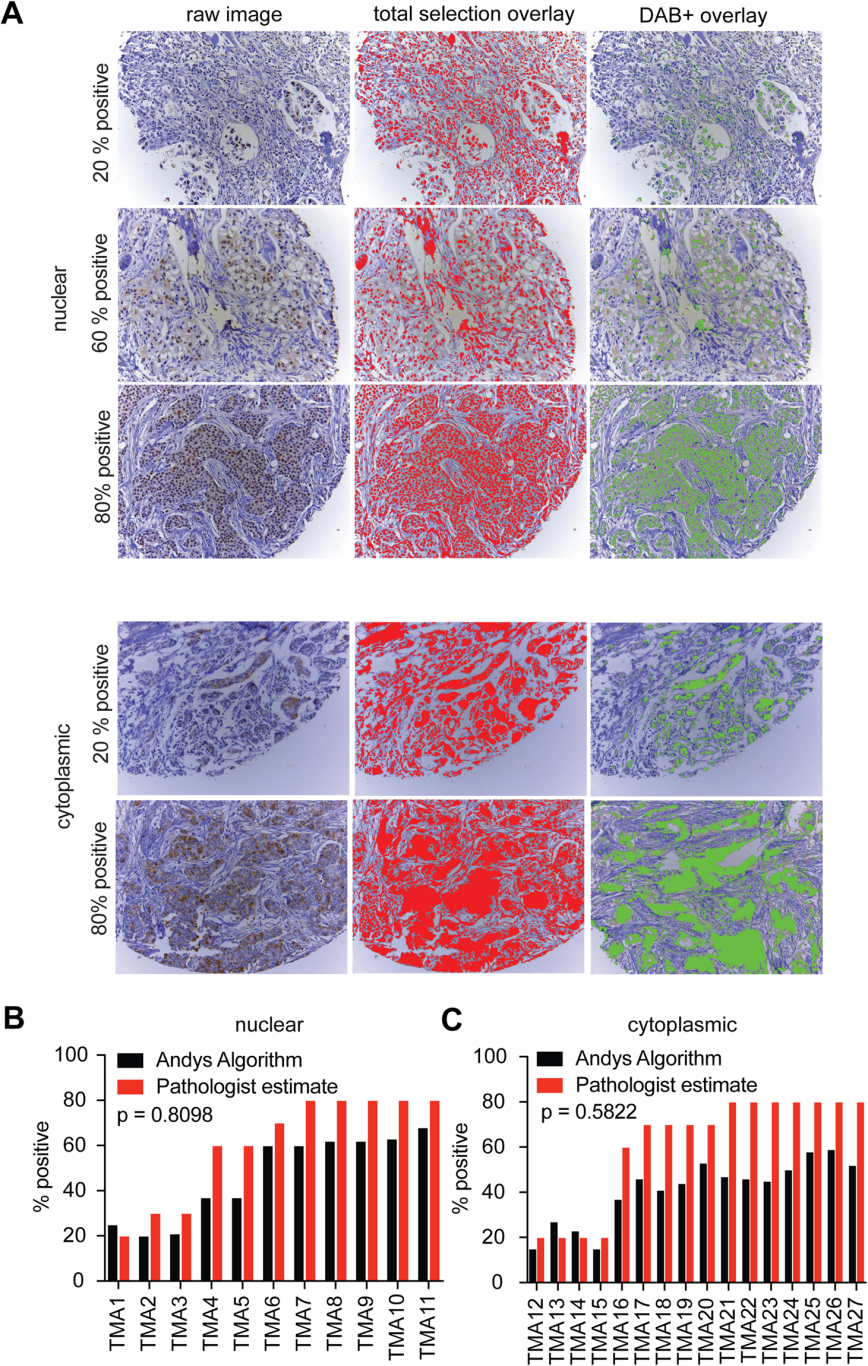
Andy’s DAB+ IHC algorithm can be used to score breast cancer TMAs. (**A**) Representative raw IHC images (left panels) depicting 20–80% nuclear or cytoplasmic expression of MCL-1 using cores selected from a cohort of TMAs in^22^. Selection overlays produced after analysis of the raw MCL-1 IHC images for total (red) and DAB+ (green) selections. Bar graphs depicting the percentage of the core positive for MCL-1 with either nuclear (**B**, n = 11**)** or cytoplasmic (**C**, n = 16**)** staining scored with either Andy’s PLA pipeline (black) or manual pathological scoring (red). Chi-squared p-value.

We have further validated Andy’s DAB+ IHC pipeline to a small cohort of human breast cancer tissue microarrays (TMAs) stained with an antibody against human myeloid cell leukemia 1 (MCL-1) from Young *et al*.^22^. MCL-1 was present in the nucleus and cytoplasm of invasive breast cancers and is expressed by luminal, basal and HER2 positive subtypes (Fig. 2A). To validate the performance of Andy’s DAB+ IHC algorithm, a subset of 27 TMAs cores was selected with invasive breast cancer showing both nuclear (11 cores) and cytoplasmic (16 cores), MCL-1 expression was compared the output of Andy’s DAB+ IHC pipeline with that of the pathologist. The percentage of the cores positive for either nuclear or cytoplasmic MCL-1 staining as determined by Andy’s DAB+ IHC pipeline corresponded well with the pathologist’s estimate (Fig. 2B, Chi squared p = 0.8098 and Fig. 2C, p = 0.5822 respectively). The pathologist estimate was frequently higher than that of the output of the DAB+ IHC pipeline and this may be due to rounding as a result of estimates that were provided in multiples of 10 by the pathologist. In contrast Andy’s DAB+ IHC algorithm was able to detect small variations in the level of staining between individual cores. Hence the Andy’s DAB+ IHC algorithm could be used to score TMAs and provide additional confirmation for the scores of a pathologist.

### A new way for performing automated image analysis of H&E immunohistochemistry and 3D colony forming assays

Using similar principles to the DAB+ image analysis algorithm, two further pipelines have been designed for particle analysis. The first is an H&E particle analysis pipeline for the discrimination of hematoxylin (H) dense regions from eosinophilic pink whole tissues in low power images (eg. lung metastatic deposits, Supp. Figure 2). The filters used in the DAB+ IHC algorithm above were also useful in discriminating the hematoxylin dense (dark blue) regions of metastases from the lighter blues and eosin pink regions of surrounding tissues and so have been employed here. The flow chart outlining the processes in this method is illustrated in Supp. Figure 2A and example images with total tissue selection and hematoxylin (H) dense regions are illustrated in Supp. Figure 2B. Similar image processing to the DAB+ IHC algorithm, including adding a Gaussian blur, conversion to an 8-bit image, manual or automatic thresholding (using the calculations Moments^33^, MaxEntropy^26^, Otsu^29^, Triangle^34^ (or Intermodes^35^ for the hematoxylin dense regions) and Yen^32^ for the selection) and particle analysis as illustrated in Supp. Figure 1 is also used in the H&E pipeline.

An additional image processing and particle analysis is provided in Andy’s 3D Colony Forming Algorithm to track growth (size), proliferation (number) and invasion (circularity) of 3D colonies *in vitro* (Supp. Figure 3A), widely used end-points for analyzing the growth of cancer cells embedded in physiologically relevant extracellular matrix^36^. Color filters in this case are not required as images are taken using bright field microscopy and converted to greyscale with image contrast to distinguish colonies from background. The Subtract Background or Normalize Local Contrast functions are required in this pipeline and are essential for removing shadowing and uneven illumination (eg. due to tissue culture wells in 3D colony forming assays, Supp. Figure 3B). Once shadowing and uneven illumination has been adjusted, the algorithm performs similar image processing to the DAB+ IHC algorithm, including adding a Gaussian blur, conversion to an 8-bit image, manual or automatic thresholding (using the calculations Huang^25^, Li^27,28^ (or MaxEntropy^26^ for the normalize local contrast selection), Otsu^29,30^, Triangle^34^, and Yen^32^) and particle analysis as illustrated in Supp. Fig. 1.

**Figure 3:**
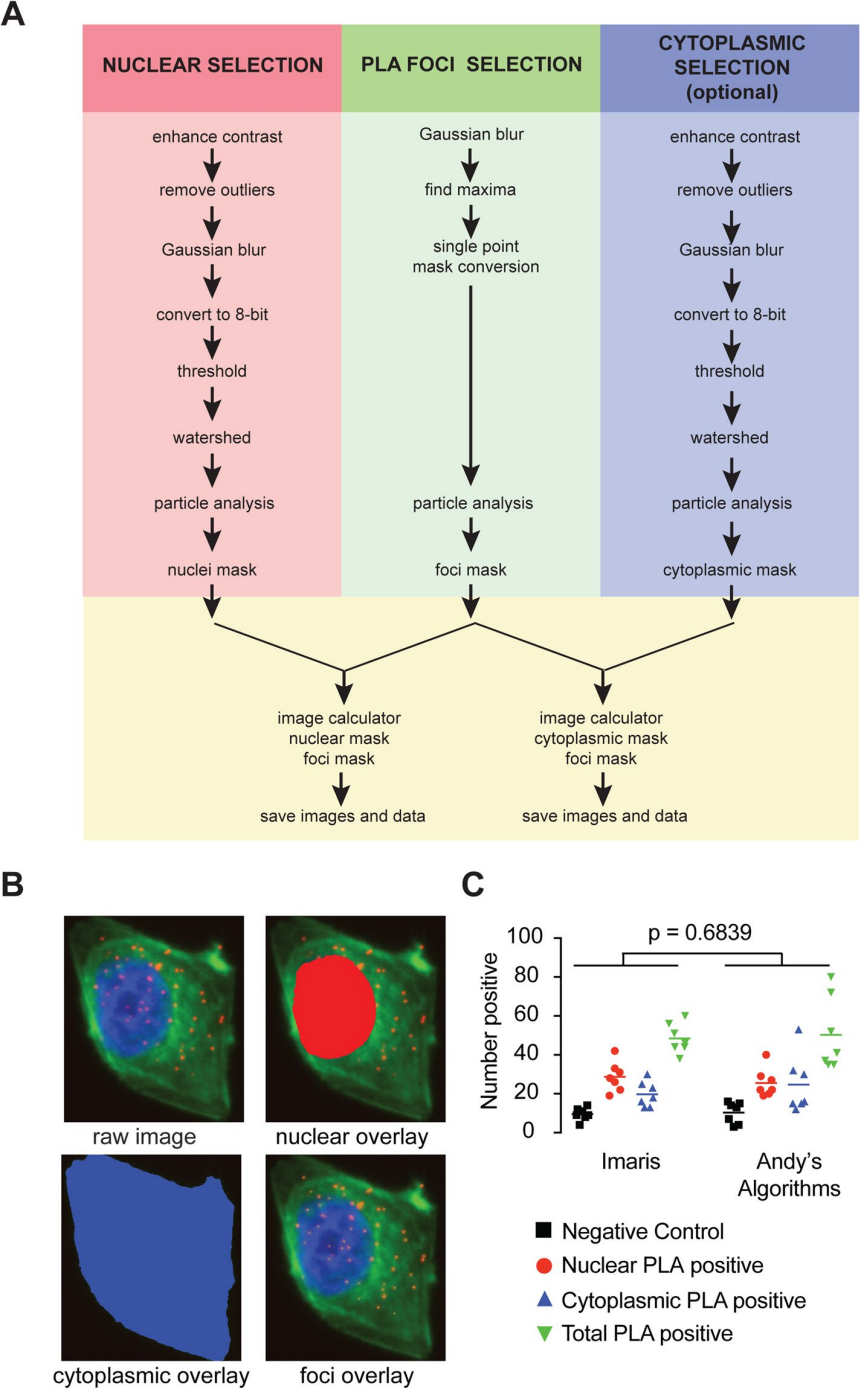
A new pipeline for image quantification for proximity ligation assays. (**A**) Flow chart depicting the image processing steps within the PLA particle analysis algorithm for the selection of all positive PLA foci. (**B**) Representative raw PLA image (top left panel) and the selection overlays for nucleus (top right, red), cytoplasm (bottom left, blue) and PLA foci (bottom right, green). (**C**) Scatter plot depicting the number of PLA positive foci within the nucleus, cytoplasm or whole cell (total) and compared to control calculated with Andy’s PLA algorithm and compared data from^37^ quantified using Imaris software (n = 7 per group, ANOVA p-value tests variance across algorithms).

### The first open-access all-in-one pipeline for image analysis of proximity ligation assays

There are currently few resources for image processing for PLAs^7^. This method is often combined with a nuclear stain and/or a cytoplasmic stain to aid analysis. We have designed a pipeline for PLAs (Andy’s PLA Algorithm) providing an all-in-one tutorial and analysis for accurate reproducible, high-throughput quantification of the total number of PLA foci in the nuclei and cytoplasm of a series of fluorescent images.

A flow chart of the PLA processing is outlined in Fig. 3A and illustrated in Supp. Fig. 4. Unlike the DAB+ IHC algorithm, each fluorescent marker for foci, nuclear and cytoplasm (optional) is acquired on a separate channel (allocated Ch00, Ch01 and Ch02). Although it is not important to acquire foci, nuclei and cytoplasm stains on any one channel, a common suffix is used for each channel so that the algorithm can allocate a specific image-processing pipeline for each condition. Like the DAB+ IHC algorithm, the PLA algorithm prompts the user to optimize a set of image parameters to fine-tune the contrast to ensure accurate image analysis. Color filters are not required to discriminate between colors in this case as each channel is dealt with separately. An enhance-contrast processing step is used on nuclei and cytoplasmic channels to overcome diffuse fluorescence. For foci selection a find-maxima function is used to identify, count and score select single foci. The remainder of the PLA pipeline operates similarly to the DAB+ IHC algorithm as detailed above and prompts the user to set a Gaussian blur to smooth the edges and reduce noise, apply a manual or automatic threshold (using the calculations Huang^25^, Intermodes^35^, Otsu^29^ and RenyiEntropy^26^) watershed to segment the image, outline regions of interest, set the scale and perform the particle analysis (as per Supp. Figure 1). The results are then provided as a single results file with new image overlay files produced for nuclei, foci, and cytoplasmic selection saved to the target directory (Fig. 3B and Supp. Fig. 4). A glossary defining the output parameters in the summary spreadsheet is provided in Supp. Table 3.

Andy’s PLA pipeline was validated against the output of the commercial image analysis software, Imaris, which was used to generate PLA interaction data published by Rogers *et al*.^37^. The known interaction of CDK2 and Cyclin E1 was examined and positive foci were observed in both the nucleus and cytoplasm of T47D breast cancer cells. The total number of nuclear, cytoplasmic and total foci as determined using Andy’s Algorithms was equivalent to that output using the commercial software Imaris (Two-way ANOVA p = 0.68). A small but insignificant variation in the cytoplasmic counts in individual images was observed and likely due to variation in cytoplasmic fluorophore intensity, which was better tolerated using Andy’s PLA pipeline (Fig. 3C). Thus, Andy’s PLA Algorithm provides the first open access, accurate, reproducible, all-in-one algorithm built for FIJI specifically designed for the analysis of PLA images for those with no experience with image analysis.

## Discussion

Accurate image analysis of large numbers of sections is essential to overcome cognitive bias and improve scientific reproducibility, which has emerged as a significant problem in pre-clinical research, particularly for informing drug development in oncology^38^. Few image analysis programs provide the wide-ranging usability to those with limited image analysis expertise^16^. Andy’s Algorithms was developed to provide all-in-one standardized, unbiased, high throughput, and reproducible image analysis pipelines for specific common biological applications (DAB+ IHC, PLA, H&E histology and 3D colony forming assays) for scientists independent of their image analysis expertise.

Each pipeline in Andy’s Algorithms prompts users to perform an image analysis optimization prior to image processing to determine an optimal set of parameters that fit best across an image cohort. This helps users tailor the pipeline for individual laboratory variation, which is dependent on, but not limited to, the cell type, tissue type, fixation method, antigen detection method, staining pattern, color discrimination, magnification, illumination settings and resolution. Andy’s Algorithms uses dialog boxes to explain and guide users through the image processing and analysis pipeline, allowing novice users to tailor the parameters in a step-by-step manner for each algorithm. The highly sensitive pipelines have been designed in a pre-clinical oncology laboratory but image analysis parameter optimization steps will facilitate wider adoption of these pipelines across laboratories and scientific disciplines providing fast, reproducible and standardized methodology for batch image quantification quantitation of DAB+ IHC, PLA, H&E histology and 3D colony forming assays.

Image analysis parameter optimization is required to overcome variations tissue processing and staining between laboratories. Andy’s Algorithms prompts users to run the image analysis optimization on a set of 3–5 images that are representative of an image set to find the best set of parameters suitable for most images in a cohort. If large variations in fixation, staining, image acquisition settings, exposure, contrast and brightness exist, then the average parameters will not perform well on outlier images in any quantification method, manual or automated. Immunohistochemistry, for example, should be performed across a cohort of samples fixed and processed in the same way, stained with the same antibody batch, and treated with the same timings and protocol in any given experiment. Furthermore images should be acquired using the same magnification, exposure, and resolution and brightness settings for the best results, for any series that will be quantified using any method. It is also preferable to perform batch image analysis on sections where antibody-antigen detection methods have been optimized to avoid saturation of chromagen or fluorophore. For example, overly dilute or concentrated DAB staining and/or hematoxylin counterstaining can lead to poorly defined nuclear outlines, resulting in difficulties in nuclei segmentation^10^. Optimal staining conditions allow for well-defined nuclei that can be easily segmented and isolated. This is also true for the analysis of PLA assays where selection of PLA foci is based on a user set value of noise tolerance in the Find Maxima function. Setting the value too low can result selecting background fluorescence and too high will exclude positives. Poor signal due to poorly optimized staining protocols will affect selection of foci and nuclear and ultimately the accuracy of batch analysis. If staining is clear and precise then minimal adjustments are required for accurate selections. We recommend that users employ any quantification technique, including Andy’s Algorithms only when fixation, staining, image acquisition settings, exposure, contrast and brightness are consistent across a cohort of samples. A troubleshooting guide has also been included at GitHub (https://github.com/andlaw1841/Andy-s-Algorithm) to assist users with overcoming these issues.

Andy’s Algorithms for FIJI^9^ offer significant benefits over existing general image analysis programs. The open source image analysis programs CellProfiler and ilastik do not provide specific image analysis pipelines for DAB+ IHC image analysis and required extensive time in developing a set of parameters for batch image analysis. Similarly, the output of Imaris, which was used to quantify PLA foci, was demanding to configure, difficult to create a batch analysis pipeline and was time consuming to compile the output data. Imaris also required an additional upgrade package to complete the process. Andy’s PLA pipeline described here is the first open-source pipeline for quantification of proximity ligation assays that could be used as a standardized assay for PLA analysis. A comparison of Andy’s Algorithms with these programs is provided in Table 1, with Andy’s pipelines providing a user-friendly, all-in-one interactive tutorial, high background sensitivity, single spreadsheet result output designed specifically for DAB+ IHC, PLA, H&E histology and 3D colony forming assay image analysis, a feature that is absent in the more general image analysis programs. Importantly Andy’s Algorithms are provided as freeware for all researchers from any field downloadable from GitHub (https://github.com/andlaw1841/Andy-s-Algorithm) facilitating further development of tailor-made applications for other specific assays using the current pipelines as backbones. Thus Andy’s Algorithms provides the first open-source all-in-one user-friendly pipelines that can be run in FIJI^9^ for high-throughput, reproducible and quantification of DAB+ IHC, PLA, H&E histology and 3D colony forming assays, which require no prior user knowledge of image analysis and that could be adopted as standardized methods for these common assays.

**Table 1:** Program comparison of Andy’s image analysis algorithms with CellProfiler, ilastik and Imaris.

	Andy’s Algorithms	CellProfiler	ilastik	Imaris
Tutorial	Interactive tutorial for each algorithm and analysis all-in-one	General program video and text tutorial	General program text tutorial	General program text tutorial
Application	Designed for specific assays for use in FIJI^9^	General image analysis program	General image analysis program	General image analysis program
Batch Analysis	✓	✓	✓	✓ (requires additional input + package upgrade)
Result output	Single summary spreadsheet output	Single summary spreadsheet output	Spreadsheet per image output - requires compilation	Spreadsheet per rendered layer/per image - requires compilation
Simplicity	Simple step-by-step method	Powerful but complex. Must design and create pipeline	Simple step-by-step method	Complex step-by-step method
Image Optimization	✓	✓	✗	✓
Overlay	✓	✓	✗	✓
Free Open Access	✓	✓	✓	✗
Signal:Background sensitivity	High	Unknown	Low	High

## Methods

### Mice

All mice were housed in specific pathogen-free conditions at the Garvan Institute, with all animal procedures approved by the Garvan/St Vincent’s Animal Ethics and Experimentation Committee (Approval #14/27). All animal experiments were performed in accordance with the NSW Animal Research Act 1985 (PDF), NSW Animal Research Legislation 2010 and the Australian code of practice for the care and use of animals for scientific purposes, 8th Edition 2013. Immune-compromised NOD.Cg-Prkdc^scid^ Il2rg^tm1Wjl^/SzJ were housed in SPF conditions in a 12-hour:12-hour light:dark cycle and given food and water ad libitum. Intraductal injections were modified from a previously described protocol without a Y incision in the abdomen^39^. For cross-sectional studies of tumour metastasis, mice were again randomized into DOX-treated or control-treated groups and sacrificed at 9 weeks (MDA-MB-231 xenografts) or 12 weeks (MDA-MB-468 xenografts) post tumor cell inoculation. Mice were then euthanized with CO_2_ asphyxiation and the lungs were harvested and fixed in 10% neutral buffered formalin for 4 hours in 10% buffered formalin at room temperature.

### Immunohistochemistry

DAB IHC validation data in Fig. 1 was performed on 4 µm lung sections from 3–4 mice bearing human breast cancer xenografts subjected to immunohistochemistry using an antibody to anti-high molecular weight cytokeratin (model 1) or anti-human vimentin (model 2) with detailed methodology described in^22^. After fixation the lungs were cut into 4 µm lung sections, baked for 4 hours at 60 °C, deparaffinised and antigens retrieved using pH9 retrieval solution (S2367) in a pressure cooker for 30 seconds. Endogenous peroxidases were blocked using a 3% H_2_O_2_ solution and then incubated with primaries antibodies against high molecular weight cytokeratin (1:100, Leica 34BE12) for lungs bearing MDA-MB-468 metastases and against Vimentin (1:400, Leica NCL-L-VIM-V9) for lungs bearing MDA-MB-231 metastases for 30 minutes at room temperature. Sections were washed and then incubated with secondary peroxidase conjugated antibody (Envision mouse K4007) before application of the DAB chromagen substrate (K3468). All reagents were from DAKO unless otherwise specified and immunohistochemistry was performed on a DAKO autostainer. 20 images from each lung bearing breast cancer metastases (n = 3–4 mice was acquired from each on a Leica DM4000 light microscope an objective of 20X and was used further image analysis. A table of the optimised image parameters used for the IHC image analysis of lung metastatic deposits is provided in Supp. Table 4.

### Breast cancer tissue microarrays

Immunohistochemistry was used to assay MCL-1 protein expression using a mouse monoclonal antibody to MCL-1 (ThermoFischer (Pierce) MA5–13932) on TMAs constructed from tumors from a cohort of 292 patients diagnosed with invasive ductal breast carcinoma described in^40^. Immunohistochemistry was performed as above except antigen retrieval (pH9 S2367) was performed in a pressure cooker for 4 minutes and primary antibody concentration was performed at a 1:20 dilution. The cohort consists of cases of invasive ductal carcinoma of no special type, median age 54 (range 24–87), with a median follow-up of 64 months (range 0–152.1). Of these, 68.6% were ER + , 57.1% were PR + , 18.7% were HER-2 amplified (by FISH) and 43.3% were lymph node-positive. Endocrine therapy (TAM) was given to 49.3% of patients and chemotherapy (AC or CMF) to 38%. MCL-1 protein could only be detected in a subset of 246 of these cases due to missing or folded cores. To examine the performance of Andy’s DAB+ IHC pipeline on this cohort, 11 cores with nuclear and 16 cores with cytoplasmic expression were randomly selected for analysis and compared to the pathologists manual estimate. A table of the optimised image parameters used for the IHC image analysis of TMAs is provided in Supp. Table 5.

### Proximity ligation assays

PLA validation data in Fig. 3 was performed in fixed cells using antibodies to CDK2 and Cyclin E1 with detailed methodology described in^37^. Cells were fixed with 4% PFA/PBS for 20 minutes at room temperature, with or without methanol post-fixation (−20 °C for 20 min). Samples were blocked with 1% BSA/PBS, stained with the indicated antibodies and counterstained with ToPro3/DAPI (Jackson ImmunoResearch Laboratories). PFA fixed cells were subjected to the Duolink Proximity Ligation Assay (Sigma) as described by the manufacturer using antibodies CDK2 (M2, D12) and cyclin E1 (HE12) (Santa Cruz Biotechnology). Confocal microscopy was performed on Leica DMRBE/DMIRE2. Images were analysed with Imaris where individual spots were defined with a variable and initial size estimate of 0.5 μm. 7 composite (foci, nuclear and cytoplasmic) images were used for image analysis. Images were processed with Adobe Photoshop, and adjusted for optimal brightness/contrast. Minimal gamma changes were made to enable visualisation of overlaid signals. A table of the optimised image parameters used for the PLA image analysis is provided in Supp. Table 6.

### Statistics

All statistics were performed in Prism7 for MacOSX. An ANOVA and a Pearson’s R^2^ correlation coefficient was used to compare between Andy’s DAB+ IHC and PLA algorithms and the general image analysis programs CellPofiler^15^, ilastik^11^ and Imaris. A Chi-squared distribution was used to determine whether there was any significant variation between the output of Andy’s DAB+ IHC and pathological scoring.

### Instructions to load Andy’s algorithms in FIJI

The image processing steps for the DAB+ IHC and PLA algorithms are illustrated in Figs 1A and 3A and Supp. Figure 1 and 4 and for the H&E and 3D Colony forming image analysis are illustrated in Supp. Figure 2A and 3A. To download, install and run Andy’s Algorithms, follow the following steps:

1. Download and install FIJI or update FIJI (version 1.51 k or later) [https://fiji.sc]
2. Download Andy’s Algorithms (eg DAB_IHC_v2.40.ijm, PLA_v2.40.ijm, HandE_v2.40.ijm and 3D_colony_v2.40.ijm) from https://github.com/andlaw1841/Andy-s-Algorithm
3. Go to Plugins > Macros > Install the Algorithm of choice in the menu bar of FIJI
4. Select the Andy’s Algorithm preference (eg DAB_IHC_v2.40.ijm) to install the algorithm
5. Go to Plugins > Macros. An option to select the algorithm will now be in the dropdown menu. Click run.

The algorithm will be temporally installed into the toolbar of FIJI and closes when FIJI is exited. Simply reinstall the algorithm when you open FIJI again. The algorithm is designed for single images and not Z-stacks. Please convert image stacks to single files before running each algorithm. Sample images are provided at https://github.com/andlaw1841/Andy-s-Algorithm to assist users with the optimization of the image processing and analysis settings.

### Data availability

The datasets generated during and/or analysed during the current study are included in this published article (and its Supplementary Information files). Andy’s DAB_IHC_2.40.ijm, PLA_2.40.ijm, HandE_2.40.ijm and 3D_colony_2.40.ijm) from https://github.com/andlaw1841/Andy-s-Algorithm. All images used in the current study will be made available at Figshare at https://figshare.com/.

## Electronic supplementary material

Supplementary Figure Legends, Tables and Figures
