# Supplementary material for: Andy’s Algorithms: new automated digital image analysis pipelines for FIJI
Source: Sci Rep. 2017 Nov 16;7:15717. doi: 10.1038/s41598-017-15885-6 (PMC5691210; doi:10.1038/s41598-017-15885-6)
Supplement: Supplementary file 1 — Supplementary Figure Legends, Tables and Figures [file 41598_2017_15885_MOESM1_ESM.pdf]

Andy's Algorithms: new automated digital image analysis pipelines for FIJI

Andrew M.K Law[1], Julia X.M Yin[1], Lesley Castillo[1], Adelaide I.J. Young[1], Catherine Piggin[1], Samuel Rogers[1], Catherine Elizabeth Caldon[1,2], Andrew Burgess[1,2], Ewan K.A. Millar[1,3,4,5], Sandra A. O'Toole[1,6], David Gallego-Ortega[1,2], Christopher J Ormandy[1,2*], and Samantha R Oakes[1,2*#]

[1] Garvan Institute of Medical Research and the Kinghorn Cancer Centre, 384 Victoria Street, Darlinghurst, NSW 2010, Australia.

[2] St. Vincent's Clinical School, UNSW Sydney, Victoria Street, NSW 2052, Australia.

[3] Department of Anatomical Pathology, South Eastern Area Laboratory Service, St George Hospital, Grey St. Kogarah, 2217, Australia.

[4] School of Medical Sciences, UNSW Sydney, Kensington, NSW 2033, Australia.

[5] School of Medicine and Health Sciences, Sydney Western University, Campbelltown, NSW 2560, Australia.

[6] Australian Clinical Labs, 112/14 Lexington Drive, Bella Vista, NSW 2153, Australia.

* Joint senior authors

# Corresponding author

Twitter @Sam_R_Oakes

Garvan Institute of Medical Research and the Kinghorn Cancer Centre. 384 Victoria Street, Darlinghurst, NSW 2010, Australia. Email: s.oakes@garvan.org.au

Telephone: 61 (2) 9355 5812 Facsimile: 61 (2) 9355 5872

Keywords: Image analysis, immunohistochemistry, 3,3′-diaminobenzidine, automated image quantification, proximity ligation assays, metastasis, intensity analysis, hematoxylin and eosin, 3D colony forming assays.

Supplementary Figure Legends

Supplementary Figure 1. Flowchart illustrating the processes in Andy's DAB+ IHC Algorithm.

A raw image is selected from the target folder and a color blindness filter (deuteranope filter for total selection or tritanope filter for DAB+ selection) is applied to enhance the selection of the region of interest (ROIs) selection prior to the application of a color deconvolution filter (Feulgen light green filter for total selection or a H&E for DAB+ selection) A Gaussian blur is then applied to the image, which is then converted to an 8-bit grey-scale image before applying a threshold. An optimal automatic threshold function is selected from five main algorithms; (Huang, RenyiEntropy (or Li for the basic pipeline), Otsu, Shanbhag, and Yen). The image is then converted to a binary image and watershed, fill holes, and edge exclusion can be applied before processing with particle exclusion. Image analysis is performed with overlay images of both the total and DAB+ selection produced in the target folder.

Supplementary Figure 2. A new pipeline for H&E image analysis

(A) Flow chart depicting the image processing steps within the H&E particle algorithm for the selection of all hematoxylin (H) rich regions within an H&E image. A raw image is selected from the target folder and a color blindness filter (deuteranope filter for total tissue selection or tritanope filter for dark blue hematoxylin selection) is applied to enhance region of interest (ROIs) selection prior to the application of a color deconvolution filter (FastRed/Fastblue/DAB filter for total tissue selection or a H&E/DAB for dark blue hematoxylin selection) to discriminate between dark blue and light blue/red. A Gaussian blur is then applied to the image, which is then converted to an 8-bit grey-scale image before applying a

threshold. An optimal automatic threshold function is applied (using the calculations Moments, MaxEntropy, Otsu, Triangle (or Intermodes for the hematoxylin dense regions) and Yen for the selection). The image is then converted to a binary image and watershed, fill holes, and edge exclusion can be applied before processing with particle exclusion. Image analysis is performed with overlay images of both the total tissue and dark blue hematoxylin dense selection produced in the target folder. **(B)** Representative raw H&E mage (left panel), the total selection overlay (middle panel) and the hematoxylin (H) dense regions (right panel).

Supplementary Figure 3. A new pipeline for 3D colony particle analysis.

(A) Flow chart depicting the image processing steps within Andy's 3D colony Algorithm for the image analysis of 3D colonies embedded in an extracellular matrix. Background is first removed from brightfield images of 3D colony forming assays. removing shadowing as a result of uneven illumination and shadowing effects (*e.g.* due to tissue culture wells in 3D colony forming assays, Supp. Fig. 3B). A Gaussian blur is then applied to the image, which is then converted to an 8-bit grey-scale image before applying a threshold. An optimal automatic threshold function is selected from five main algorithms; Huang, Li (or MaxEntropy for the normalize local contrast selection), Otsu, Triangle, and Yen). The image is then converted to a binary image and waterhshed, fill holes, and edge exclusion can be applied before processing with particle exclusion. Image analysis is performed with overlay images of both the total tissue and dark blue hematoxylin dense selection produced in the target folder.

Supplementary Figure 4. Flowchart outlining Andy's PLA Algorithm used in the series analysis. Raw image is identified within the folder and opened in FIJI. The

nuclei image is processed first, followed by the foci image, and finally by the cytoplasmic image. Nuclei and cytoplasmic images are processed with Enhanced Contrast and Remove Outliers before it is converted to an 8-bit grey scale image. A maxima selection with an adjustable noise tolerance is used to identify and select the signals in the foci image. A manual or automatic threshold is set (using the calculations Huang, Intermodes, Otsu and RenyiEntropy) for nuclei and cytoplasmic images are automatically then converted to a binary mask image. Mask conversion of foci image is based on maxima selection. Final selection is overlaid on top of the raw image and nuclei and cytoplasmic foci signals are differentiated. A more detailed explanation of the processes and additional steps are outlined within the tutorial of the PLA algorithm.

Supplementary Table 1: List of open access and commercial image processing and analysis programs, website and references.

Name	Website	Reference
Open-Access Programs		
ImageJ	https://imagej.nih.gov/ij/	[1]
FIJI	https://fiji.sc/	[2]
ImmunoRatio	http://153.1.200.58:8080/immunoratio/	[3]
CellProfiler	http://cellprofiler.org	[4]
ilastik	ilastik.org/	[5]
Icy	icy.bioimageanalysis.org/	[6]
Daime	dome.csb.univie.ac.at/daime	[7]
BlobFinder	www.cb.uu.se/~amin/BlobFinder/	[8]
VIGRA (Vision with Generic Algorithms)	https://ukoethe.github.io/vigra/	
Commercial Algorithms		
MATLAB	https://www.mathworks.com/products/matlab.html	
MetaMorph	https://www.moleculardevices.com/systems/metamorph-research-imaging/metamorph-microscopy-automation-and-image-analysis-software	
Duolink® ImageTool	http://www.sigmaaldrich.com/catalog/product/sigma/duo90806?lang=en&region=AU	
Imaris	http://www.bitplane.com/imaris/imaris	

Supplementary Table 2. Recommended size exclusion parameters for different magnification.

**Magnification**	**Lower Size Exclusion (pixel size)**	**Upper Size Exclusion (pixel size)**
10x	40	infinity
20x	100	infinity
40x	150	infinity

Supplementary Table 3. Glossary defining the output parameters in the summary spreadsheet for the DAB+ IHC, PLA, H&E and 3D colony forming assay pipelines.

IHC Glossary

Average Intensity	The average mean grey value of all positive ROI, that ranges from 0-255 where 0 is darkest (black) and 255 is brightest (white).
Percent Area	Percentage DAB positive area relative to the total area measured (Area of positive selection divided by area of total selection multiplied by 100)
Percent Count	Percentage DAB positive count relative to the total count measured (Count of positive selection divided by count of total selection multiplied by 100)
Positive Area	The area of the DAB positive selection (positive ROI), which can be visualized in the "positive selection mask" image
Positive Count	The count of the DAB positive selection (positive ROI), which can be identified in the "positive ROI" zip file
Positive Mask Image	Black and white binary image where the positive count and area is measured from
Positive Overlay Image	Pseudo-color image with the positive ROI overlaid on top of the raw image
ROI	Region of Interest
Total Area	The area of the total selection (total ROI), which can be visualized in the "total selection mask" image
Total Count	The count of the total selection (total ROI), which can be identified in the "total ROI" zip file
Total Mask Image	Black and white binary image where the total count and area is measured from
Total Overlay Image	Pseudo-color image with the total ROI overlaid on top of the raw image

PLA Glossary

Average Cytoplasmic Area	The average area of each cytoplasm based on the number of nuclei identified (cytoplasm area divided by nucleus count)
Average Nuclei Area	Average area of each nuclei (total nuclei area divided by nucleus count)
Average Signal per Cytoplasm	Average foci in each cytoplasmic region (cytoplasmic signal divided by nucleus count)
Average Signal per Nucleus	Average foci in each nucleus (nuclear signal divided by nucleus count)
Cytoplasm Area	The total area of all cytoplasmic region measured from the "cytoplasm mask" image
Cytoplasm Mask	Black and white binary image where the total cytoplasmic area is measured from

Cytoplasm Overlay	Pseudo-color image with the cytoplasmic ROI overlaid on top of the raw cytoplasm image
Cytoplasmic Signal	Total number of foci identified within cytoplasmic regions
Exctracellular Signal	Total number of foci identified outside of both nuclear and cytoplasmic region (total signal minus intracellular signal)
Foci Overlay	Pseudo-color image with the foci ROI overlaid on top of the raw foci image
Intracellular Signal	Total number of foci identified within either nuclear or cytoplasmic regions (nuclear signal plus cytplasmic signal)
Non Cytoplasmic Signal	Total number of foci identified outside of cytoplasmic regions
NonNuclear Signal	Total number of foci identified outside of all nuclei
Nuclear Signal	Total number of foci identified within all nuclei
Nuclei Mask	Black and white binary image where the total nuclei area and count is measured from
Nuclei Overlay	Pseudo-color image with the nuclear ROI overlaid on top of the raw nucleus image
Nucleus Count	Total number of nuclei identified in the image based on the "nuclei ROI" zip file
Percent Cytoplasmic Signal	Percentage of total foci that are cytoplasmic (cytoplasmic signal divided by total signal multiplied by 100)
Percent Nuclear Signal	Percentage of total foci that are nuclear (nuclear signal divided by total signal multiplied by 100)
ROI	Region of Interest
Total Nuclei Area	Total area of all nuclei measured from the "nuclei mask" image
Total Signal	Total number of foci identified, which can be identified in the "all foci ROI" zip file

H&E Glossary

Percent Area	Percentage hematoxylin positive area relative to the total area measured (Area of positive selection divided by area of total selection multiplied by 100)
Percent Count	Percentage hematoxylin positive count relative to the total count measured (Count of positive selection divided by count of total selection multiplied by 100)
Positive Area	The area of the hematoxylin positive selection (positive ROI), which can be visualized in the “positive selection mask" image
Positive Count	The count of the hematoxylin positive selection (positive ROI), which can be identified in the "positive ROI" zip file
Positive Mask Image	Black and white binary image where the positive count and area is measured from
Positive Overlay Image	Pseudo-color image with the positive ROI overlaid on top of the raw image

ROI	Region of Interest
Total Area	The area of the total selection (total ROI), which can be visualized in the “total selection mask" image
Total Count	The count of the total selection (total ROI), which can be identified in the "total ROI" zip file
Total Mask Image	Black and white binary image where the total count and area is measured from
Total Overlay Image	Pseudo-color image with the total ROI overlaid on top of the raw image

3D Colony Assay Glossary

Average Area Per Colony	The average area of each colony (total area of all colony divided by colony counts)
Average Aspect Ratio Per Colony	The average aspect ratio of each colony based on the major axis divided by the minor axis
Average Circularity Per Colony	The average circularity of each colony that ranges from 0-1 where 1 is a perfect circle and 0 is an elongated polygon
Cell Mask	Black and white binary image where the total colony count and area is measured from
Cell Overlay	Pseudo-color image with the total ROI overlaid on top of the raw image
Colony Counts	The count of total number of colonies, which can be identified in the "cells ROI" zip file
ROI	Region of Interest
Total Area of All Colony	The area of the total selection, measured based on the "cell masks" image

Supplementary Table 4. Optimized parameters used in the analysis of DAB+ IHC lung metastasis

IHC Model 1 Analysis Parameters

IHC Optimization	Optimization
Total Selection	Enhanced
Positive Selection	Basic
Threshold	Manual
Total Gaussian Blur	0
Total Lower Threshold	0
Total Upper Threshold	190
Total Lower Size Exclusion	50
Total Upper Size Exclusion	infinity
Total Lower Circularity Exclusion	0
Total Upper Circularity Exclusion	1
Total Watershed	FALSE
Total Exclusion	FALSE
Positive Gaussian Blur	3
Positive Lower Threshold	0
Positive Upper Threshold	120
Positive Lower Size Exclusion	200
Positive Upper Size Exclusion	infinity
Positive Lower Circularity Exclusion	0
Positive Upper Circularity Exclusion	1
Positive Watershed	FALSE
Positive Exclusion	FALSE

IHC Model 2 Analysis Parameters

IHC Optimization	Optimization
Total Selection	Enhanced
Positive Selection	Basic
Threshold	Manual
Total Gaussian Blur	0
Total Lower Threshold	0
Total Upper Threshold	210
Total Lower Size Exclusion	50
Total Upper Size Exclusion	infinity
Total Lower Circularity Exclusion	0
Total Upper Circularity Exclusion	1
Total Watershed	FALSE
Total Exclusion	FALSE
Positive Gaussian Blur	3
Positive Lower Threshold	0
Positive Upper Threshold	185
Positive Lower Size Exclusion	200
Positive Upper Size Exclusion	infinity
Positive Lower Circularity Exclusion	0
Positive Upper Circularity Exclusion	1
Positive Watershed	FALSE
Positive Exclusion	FALSE

IHC Images Corrected For Background

IHC Optimization	Optimization
Total Selection	Enhanced
Positive Selection	Basic
Threshold	Manual
Total Gaussian Blur	0
Total Lower Threshold	0
Total Upper Threshold	175
Total Lower Size Exclusion	150
Total Upper Size Exclusion	infinity
Total Lower Circularity Exclusion	0
Total Upper Circularity Exclusion	1
Total Watershed	FALSE
Total Exclusion	FALSE
Positive Gaussian Blur	0
Positive Lower Threshold	0
Positive Upper Threshold	90
Positive Lower Size Exclusion	200
Positive Upper Size Exclusion	infinity
Positive Lower Circularity Exclusion	0
Positive Upper Circularity Exclusion	1
Positive Watershed	FALSE
Positive Exclusion	FALSE

Supplementary Table 5. Optimized parameters used in the analysis of in the analysis of TMA series.

IHC Optimization	Optimization
Total Selection	Enhanced
Positive Selection	Enhanced
Threshold	Manual
Average Total Gaussian Blur	2
Average Total Lower Threshold	0
Average Total Upper Threshold	130
Average Total Lower Size Exclusion	100
Average Total Upper Size Exclusion	infinity
Average Total Lower Circularity Exclusion	0
Average Total Upper Circularity Exclusion	1
Total Watershed	FALSE
Total Edge Exclusion	FALSE
Total Fill Holes	FALSE
Average Positive Gaussian Blur	2
Average Positive Lower Threshold	0
Average Positive Upper Threshold	125
Average Positive Lower Size Exclusion	100
Average Positive Upper Size Exclusion	infinity
Average Positive Lower Circularity Exclusion	0
Average Positive Upper Circularity Exclusion	1
Positive Watershed	FALSE
Positive Edge Exclusion	FALSE
Positive Fill Holes	FALSE

Supplementary Table 6. Optimized parameters used in the analysis of in the analysis of PLA series.

PLA Optimization	Optimization
Nuclei Unique Name	Nuc
Foci Unique Name	Foci
Cytoplasm Unique Name	Cyto
Threshold	Automatic
Nucleus Bright Radius	5
Nucleus Bright Threshold	50
Nucleus Dark Radius	5
Nucleus Dark Threshold	50
Nucleus Gaussian Blur	2
Nucleus Lower Threshold	Auto=Huang
Nucleus Upper Threshold	NaN
Nucleus Lower Size Exclusion	5000
Nucleus Upper Size Exclusion	infinity
Nucleus Lower Circularity Exclusion	0
Nucleus Upper Circularity Exclusion	1
Nucleus Watershed	FALSE
Nucleus Exclude	FALSE
Foci Maxima Value	4
Cytoplasm Bright Radius	0
Cytoplasm Bright Threshold	0
Cytoplasm Dark Radius	0
Cytoplasm Dark Threshold	0
Cytoplasm Gaussian Blur	2
Cytoplasm Lower Threshold	Auto=Huang
Cytoplasm Upper Threshold	NaN
Cytoplasm Lower Size Exclusion	5000
Cytoplasm Upper Size Exclusion	infinity
Cytoplasm Lower Circularity Exclusion	0
Cytoplasm Upper Circularity Exclusion	1
Cytoplasm Watershed	FALSE
Cytoplasm Exclude	FALSE

Supplementary Information

Supplementary References

1 Schneider, C. A., Rasband, W. S. & Eliceiri, K. W. NIH Image to ImageJ: 25 years of image analysis. *Nat Methods* **9**, 671-675 (2012).

2 Schindelin, J. *et al.* Fiji: an open-source platform for biological-image analysis. *Nat Methods* **9**, 676-682, doi:10.1038/nmeth.2019 (2012).

3 Tuominen, V. J., Ruotoistenmaki, S., Viitanen, A., Jumppanen, M. & Isola, J. ImmunoRatio: a publicly available web application for quantitative image analysis of estrogen receptor (ER), progesterone receptor (PR), and Ki-67. *Breast Cancer Res* **12**, R56, doi:10.1186/bcr2615 (2010).

4 Carpenter, A. E. *et al.* CellProfiler: image analysis software for identifying and quantifying cell phenotypes. *Genome Biol* **7**, R100, doi:10.1186/gb-2006-7-10-r100 (2006).

5 Sommer, C., Strähle, C., Köthe, U. & Hamprecht, F. A. in *Eighth IEEE International Symposium on Biomedical Imaging (ISBI)* 230-233 (2011).

6 de Chaumont, F. *et al.* Icy: an open bioimage informatics platform for extended reproducible research. *Nat Methods.* **9**, 690-696. doi: 610.1038/nmeth.2075. (2012).

7 Daims, H., Lucker, S. & Wagner, M. daime, a novel image analysis program for microbial ecology and biofilm research. *Environ Microbiol.* **8**, 200-213. (2006).

8 Allalou, A. & Wahlby, C. BlobFinder, a tool for fluorescence microscopy image cytometry. *Comput Methods Programs Biomed.* **94**, 58-65. doi: 10.1016/j.cmpb.2008.1008.1006. Epub 2008 Oct 1023. (2009).

Supplementary Information

Supplementary Figures

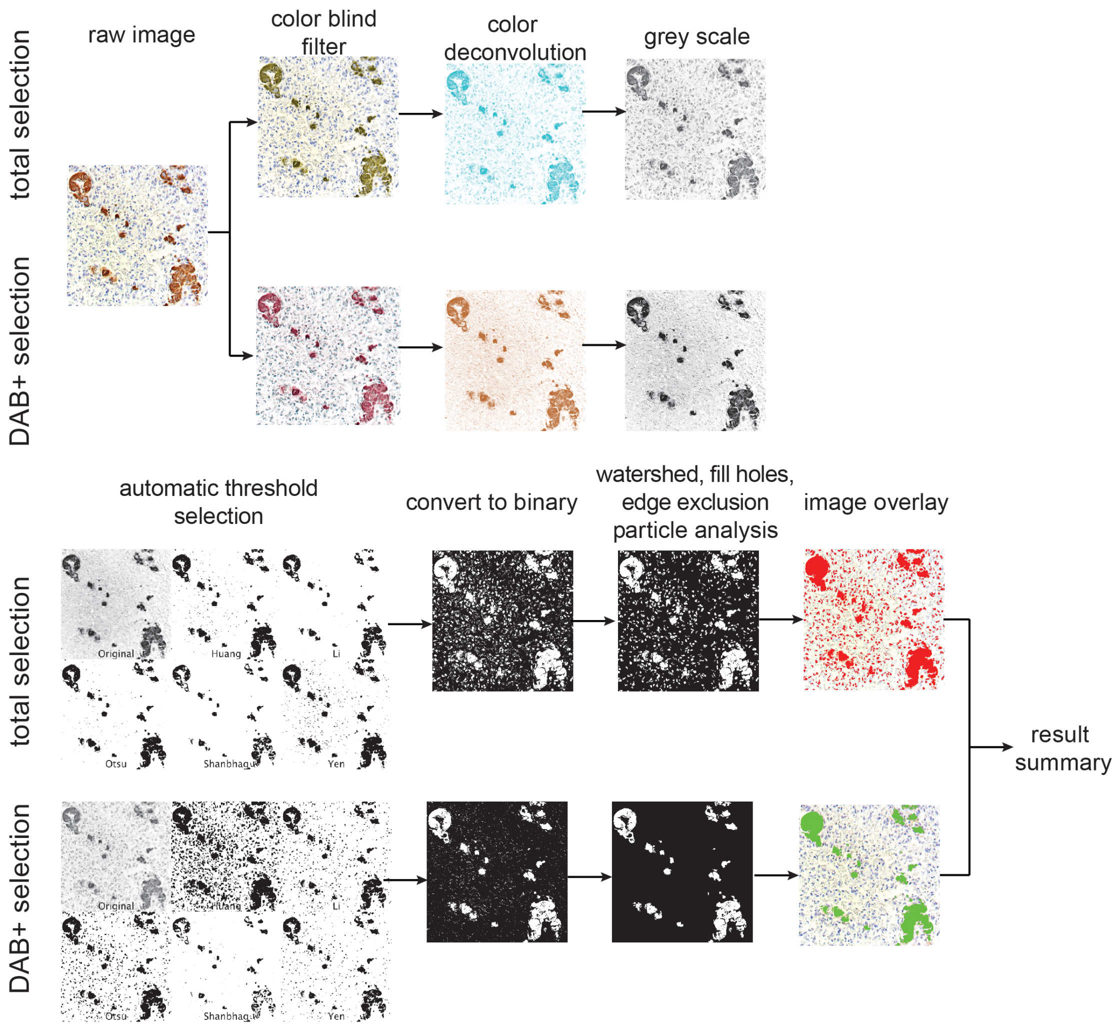

Law *et al* Supplementary figure 1

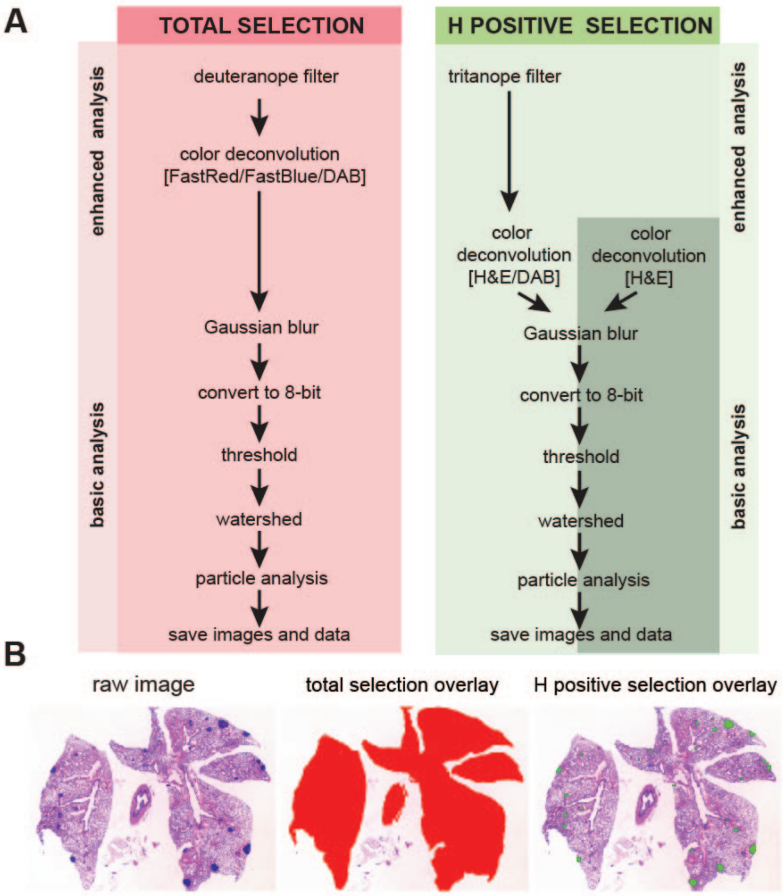

Law *et al* Supplementary Figure 2

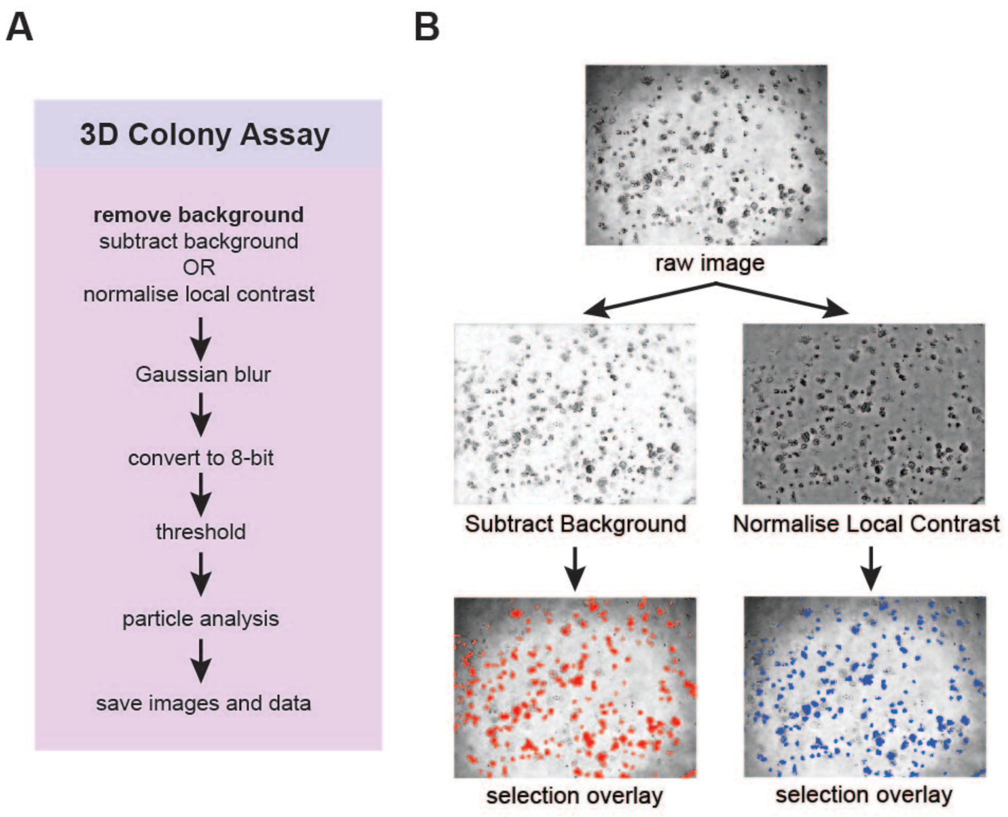

Law *et al* Supplementary figure 3

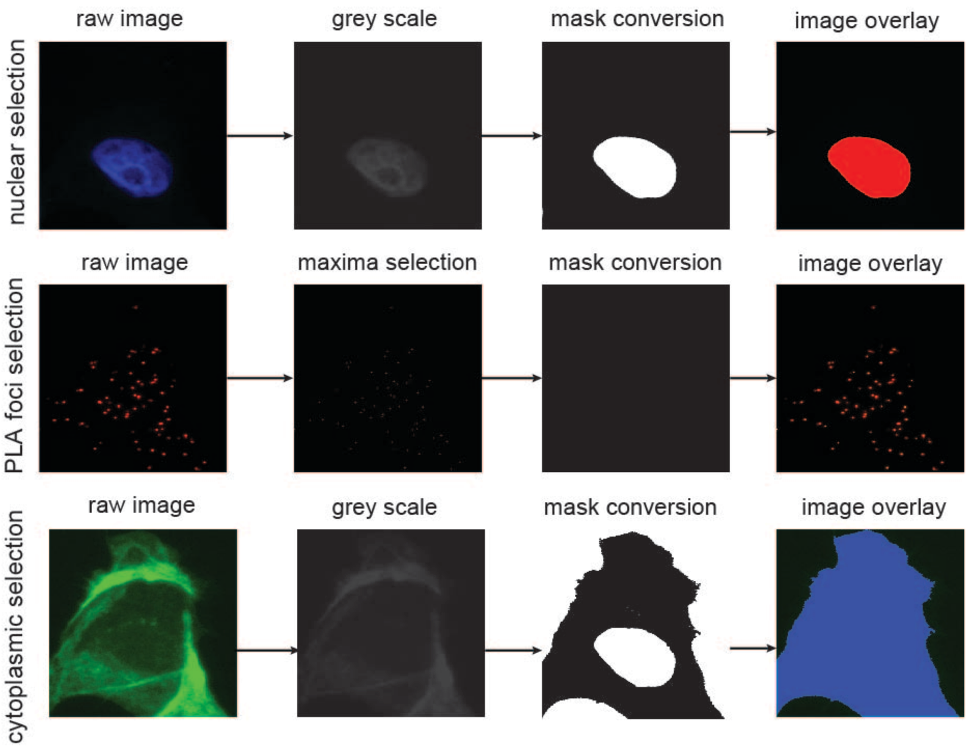

Law *et al* Supplementary figure 4
